# Bleeding Mystery Unveiled: A Case of Acquired Hemophilia A in the Shadow of Multiple Myeloma

**DOI:** 10.7759/cureus.65330

**Published:** 2024-07-25

**Authors:** Hemanthkumar Athiraman, Mani Maheshwari, Clayton Polowy

**Affiliations:** 1 Hospital Medicine, Banner Health, Phoenix, USA; 2 Hematology and Medical Oncology, Banner Health, Phoenix, USA

**Keywords:** monoclonal gammopathy of undetermined significance (mgus), hematology-oncology, stem-cell transplant, diagnosis of multiple myeloma, acquired factor viii deficiency, secondary autoimmune diseases, radiation and clinical oncology

## Abstract

This case report is of a 50-year-old woman who had a working diagnosis of von Willebrand disease (vWD) due to a history of bleeding complications and continued to experience recurrent bleeding incidents and hematoma. A workup revealed multiple lytic lesions, and a bone marrow biopsy yielded the diagnosis of multiple myeloma. After stem cell transplantation, the patient's factor VIII levels normalized, supporting acquired factor VIII deficiency due to an autoimmune phenomenon. This case highlights the rare occurrence of acquired factor VIII deficiency secondary to multiple myeloma. It also emphasizes the importance of considering secondary causes in patients with a working diagnosis of vWD and recurrent bleeding incidents.

## Introduction

Factor VIII deficiency usually presents as excessive bleeding; it can be congenital or acquired. Congenital hemophilia usually presents in the first few months of life to childhood, depending on severity, and occurs with a known family history [1]. Acquired hemophilia, on the other hand, can occur at any point in one’s life due to autoantibodies against factor VIII. Acquired hemophilia has a high morbidity and mortality rate, requiring effective and prompt diagnosis and treatment [2]. The causes of acquired hemophilia are autoimmune diseases, postpartum states, cancers, medications, and viral infections [3].

Acquired hemophilia is rare, occurring at 1.5 per million per year, making the diagnosis quite challenging. After a thorough evaluation, the cornerstone of management is bleeding control, replacing factor VIII, and patient education [3]. This life-threatening disease should be managed by a multidisciplinary team in the hospital [4].

## Case presentation

A 50-year-old female presented with postoperative hematoma, requiring evacuation and correction of coagulopathy. Her history includes previous bleeding complications after surgery, which required evacuation of the bleeding and drainage tubes through which she continued to bleed. At that time, she was noted to have prolonged partial thromboplastin time (PTT), von Willebrand factor (vWF) antigen of less than 15%, and a factor VIII inhibitor of 0.0. Factors V, VII, IX, X, XI, XII, and XIII were within normal limits. She was diagnosed with von Willebrand disease (vWD, Table 1) and was treated with Humate-P with correction of her PTT, and she was discharged soon after. She went on to get Humate-P four more times after procedures, including dental surgery (Humate-P replaces vWF and factor VIII, used to treat vWD). She was diagnosed with acquired vWD secondary to monoclonal gammopathy.

**Table 1:** Laboratory data PT: prothrombin time; INR: international normalized ratio; APTT: activated partial thromboplastin time; vWF: von Willebrand factor; SPEP: serum protein electrophoresis; IFES: immunofixation electrophoresis; IgG: immunoglobulin G; IgA: immunoglobulin A; IgM: immunoglobulin M

Laboratory	Value		Normal range
PT	10.1 second(s)		10-13 seconds
INR	1.0		0.8-1.1
APTT	38 second(s) (high)		25-36 seconds
Thrombin time	14.9 second(s)		12-19 seconds
Fibrinogen activity	375 mg/dL		200-400 mg/dL
Fibrin degradation products	Abnormal		Normal
Platelets	523 K/MM3 (high)		150-450 K/MM3
Factor V activity	123%		50-200%
Factor VII activity	106%		50-200%
Factor VIII activity	20 % (low)		50-150%
Factor VIII inhibitor	0.0		N/A
Factor IX activity	114%		50-150%
Factor X activity	106 %		76-183 %
Factor XI activity	80%		65-130%
Factor XII activity	96%		50-200%
vWF antigen	<15%		60-160%
vWF activity	<10% (low)		50-150%
Beta-2-microglobulin		1.9 mg/L	1.5-3 mg/L
IgG		1900 mg/dL (high)	600-1700 mg/dL
IgA		16 mg/dL (low)	70-400 mg/dL
IgM		28 mg/dL (low)	40-230 mg/dL
SPEP			
Kappa light chain, free		40.88 mg/L (high)	0.33-1.94 mg/dL
Lambda light chain, free		6.93 mg/L (high)	0.57-2.63 mg/dL
Kappa/lambda, free		5.90 (high)	0.26-1/65
IFES		Monoclonal gammopathy, IgG, kappa type	

She underwent Mohs surgery for a lesion on her nose and a resection of a lesion on her chest. She was treated with Humate-P multiple times, pre- and post-operatively. Initially, the patient was “doing fine.” However, the patient noticed increased facial swelling and chest discomfort two days later. This progressed, and the patient’s husband insisted that she come in to see the surgeon earlier than expected. During this visit, the surgeon found significant swelling in the nasal region and chest and immediately requested inpatient admission and additional Humate-P.

On a physical exam, the patient’s vital signs were afebrile, with a temperature of 37.1°C, a heart rate of 80, a blood pressure of 115/705, an oxygen saturation of 100% on room air, and a respiratory rate of 16. On a physical exam, ecchymoses were noted on the face and chest - darkish purple and lighter yellow tones, consistent with new and old bruising. Pertinent laboratory assessment included a white blood cell count of 8,900 cells/uL (normal range is 4,5000 to 11,000 cells/uL), hemoglobin of 10.8 g/dL (normal range is 12.1 to 15.2 g/dL), platelets of 249,000 platelets/uL (normal range is 150,000 to 450,000 platelets/uL), and activated PTT (APTT) of 50 seconds (normal range is 25 to 35 seconds). The patient received two doses of Humate-P, followed by IVIG and Gamunex. APTT normalized and remained normal for the next 24 hours until she was discharged.

**Figure 1:**
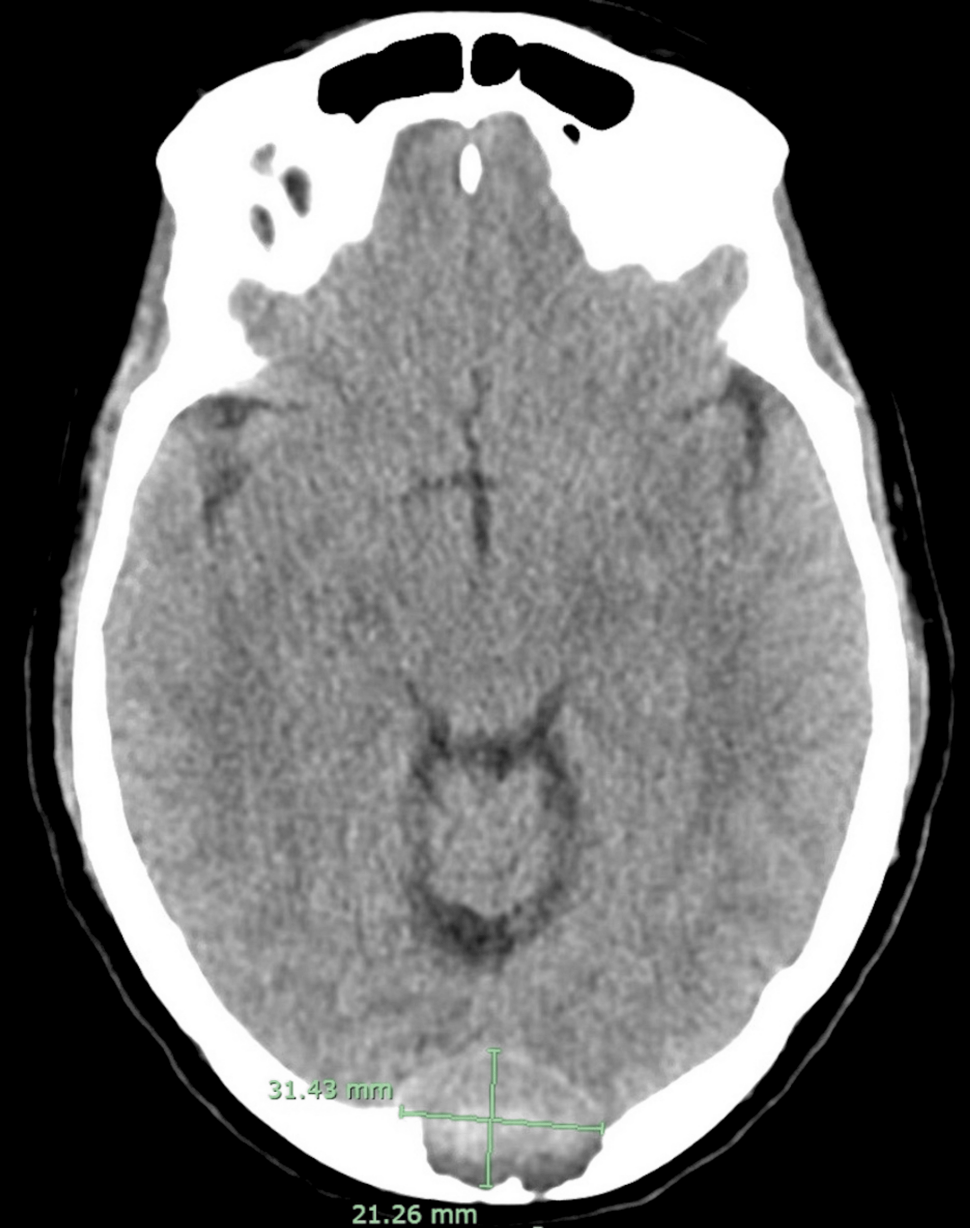
CT shows a heterogeneous extra-axial mass demonstrated at the level of the torcula and appeared to be displacing the dural venous sinuses anteriorly. The mass causes nearly full-thickness destruction of the occipital bone. The mass measures 2.1 x 3.1 x 2.8 cm in diameter. CT: computed tomography

**Figure 2:**
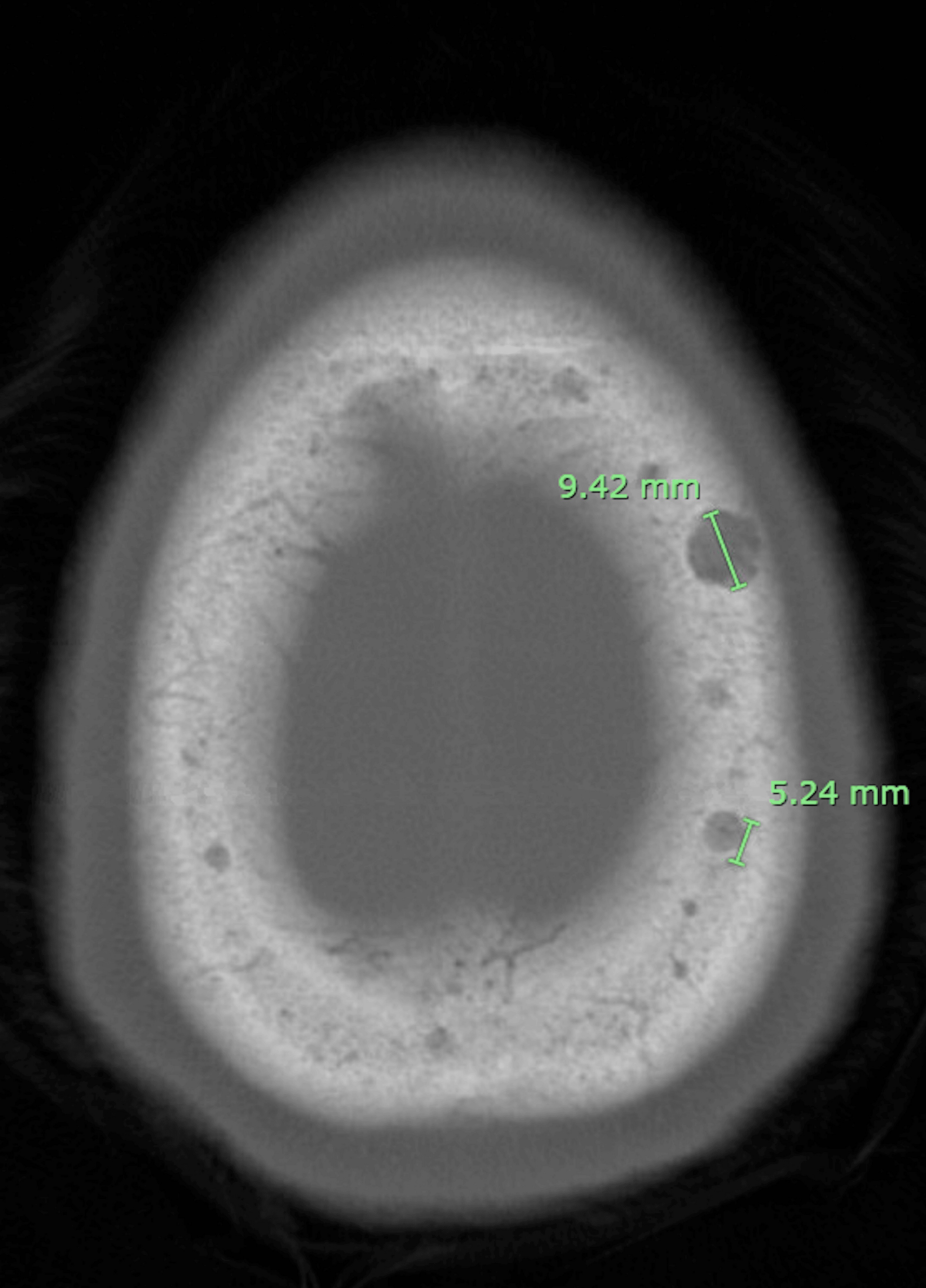
CT shows lytic lesions present bilaterally in the calvarium near the calvarial apex. The largest is on the right side, measuring 9.42 mm in diameter. CT: computed tomography

Many years later, she was hospitalized due to weakness, difficulty walking, fatigue, neck pain, and right shoulder pain. CT imaging was done, and multiple lytic lesions were revealed (Figures 1-2).

**Figure 3:**
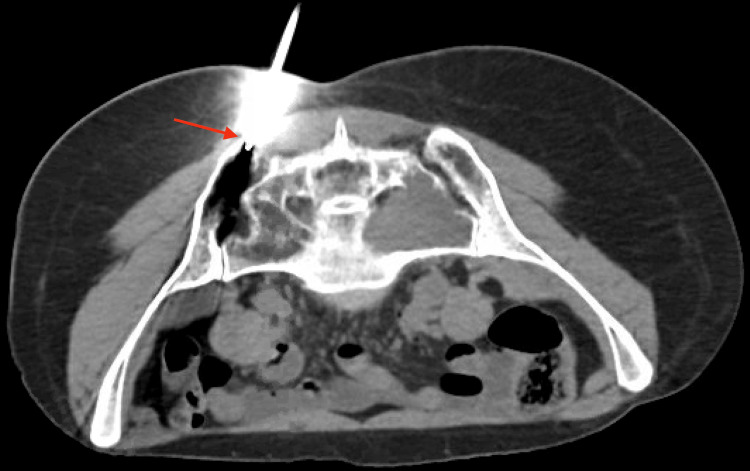
Red arrow shows CT-guided bone marrow biopsy and aspiration. CT: computed tomography

The patient was admitted, treated with analgesics and dexamethasone, and underwent a CT-guided bone marrow biopsy and aspiration after an intravenous immunoglobulin (IVIG) infusion (Figure 3). The left iliac crest bone marrow biopsy results showed the presence of phenotypically abnormal plasma cells, consistent with a plasma cell neoplasm. Chromosome analysis showed Monosomy 13, concordant with the concurrent FISH testing, a non-random abnormality seen in plasma cell myeloma.

The patient underwent radiation, followed by chemotherapy. Subsequently, she underwent a stem cell transplant as treatment for multiple myeloma. With time, the patient’s factor VIII level and vWF activity normalized. This observation indeed revealed the patient’s correct diagnosis of acquired factor VIII deficiency due to autoantibodies created secondary to multiple myeloma.

## Discussion

Acquired hemophilia (factor VIII deficiency) can be an autoimmune phenomenon caused by B cell hyperactivity in multiple myeloma. In this case, the normalization of factor VIII levels post-stem cell transplant proves this. It is now well known that B cells steer the autoimmune pathway by presenting native proteins to T cells. This B cell hyperactivity results in sustained immune stimulation, which could be a possible link; however, the mechanism is poorly understood to date. Another possible link is that during this hyperactive state, defective receptor editing can lead to B cells escaping the secondary screening before being released from the bone marrow [5].

If a B cell has a receptor for self-antigens, then receptor editing can result in successful editing so that the B cell no longer has receptors that recognize native proteins, helping these B cells to survive in the long run. The B cells that remain despite an attempt at receptor editing usually die of apoptosis but, at rare times, can escape the bone marrow and cause autoimmune cascades [5].

Hence, one of the diseases that can occur due to this B cell hyperactivity and escape from the bone marrow is acquired hemophilia. The most common causes of acquired hemophilia are pregnancy, malignancy, and infections. Hence, in diagnosing and treating patients with hemophilia, it is essential for physicians to include protein electrophoresis, immunofixation, and analysis of free light chains in their evaluation [6]. Aside from bone marrow biopsy and FISH testing (Monosomy 13 is a worse prognosis), the conventional labs to evaluate for vWD and factor VIII deficiency (hemophilia A) are vWF antigen and activity, factor VIII activity, ristocetin cofactor test, and factor VIII binding assay [7]. Since vWF binds to factor VIII, it prevents its breakdown. Hence, vWD and hemophilia A can occur together if there are low levels of vWF, showing a prolonged APTT [8]. Due to this, basic labs such as complete blood count, bleeding time, prothrombin time, and APTT should also be collected.

To date, no definitive link between cancer and the formation of antibodies to factor VIII has been established. However, the literature has noted that treating the cancer eliminates the antibodies [9]. One such case exists in the literature, which shows regression of acquired hemophilia A after chemotherapy-induced remission of multiple myeloma [10]. Similarly, the patient in this case was relieved of acquired hemophilia A after treating multiple myeloma using radiation therapy, chemotherapy, and stem cell transplantation.

## Conclusions

In a patient with a history of bleeding complications, it is important for healthcare providers to thoroughly consider secondary causes for acquired factor VIII deficiency. This case report underscores a rare autoimmune phenomenon associated with multiple myeloma, leading to the production of autoantibodies that obstruct factor VIII. While acquired hemophilia A due to multiple myeloma is exceptionally rare, diagnosing this condition requires a comprehensive approach. Necessary tests include a complete blood count, bleeding time, prothrombin time, APTT, serum protein electrophoresis, bone marrow biopsy, and FISH testing. Undertaking these tests is crucial to confirming the diagnosis of acquired hemophilia A due to multiple myeloma.

## References

[1] Salen P, Babiker HM (2023). Hemophilia A. StatPearls [Internet].

[2] Haider MZ, Anwer F (2023). Acquired hemophilia. StatPearls [Internet].

[3] Huth-Kühne A, Baudo F, Collins P (2009). International recommendations on the diagnosis and treatment of patients with acquired hemophilia A. Haematologica.

[4] Pishko AM, Doshi BS (2022). Acquired hemophilia A: Current guidance and experience from clinical practice. J Blood Med.

[5] Ananya JM, Shoenfeld Y, Rojas-Villarraga A, Levy RA, Cervera R (2013). Autoimmunity: from bench to bedside. https://www.ncbi.nlm.nih.gov/books/NBK459447/.

[6] Jalowiec KA, Andres M, Taleghani BM (2020). Acquired hemophilia A and plasma cell neoplasms: a case report and review of the literature. J Med Case Rep.

[7] Binder M, Rajkumar SV, Ketterling RP (2017). Prognostic implications of abnormalities of chromosome 13 and the presence of multiple cytogenetic high-risk abnormalities in newly diagnosed multiple myeloma. Blood Cancer J.

[8] Castaman G (2011). Treatment of von Willebrand disease with FVIII/VWF concentrates. Blood Transfus.

[9] Sallah S, Wan JY (2001). Inhibitors against factor VIII in patients with cancer. Analysis of 41 patients. Cancer.

[10] Innao V, Allegra A, Morreale R, Russo S, Musolino C (2017). Disappearance of acquired hemophilia A after complete remission in a multiple myeloma patient. Turk J Haematol.

